# Direct Stimulation of Islet Insulin Secretion by Glycolytic and Mitochondrial Metabolites in KCl-Depolarized Islets

**DOI:** 10.1371/journal.pone.0166111

**Published:** 2016-11-16

**Authors:** Javier Pizarro-Delgado, Jude T. Deeney, Barbara E. Corkey, Jorge Tamarit-Rodriguez

**Affiliations:** 1 Obesity Research Center, Department of Medicine, Boston University School of Medicine, Boston, MA, United States of America; 2 Biochemistry Department, Medical School, Complutense University, Madrid, Spain; NIDCR/NIH, UNITED STATES

## Abstract

We have previously demonstrated that islet depolarization with 70 mM KCl opens Cx36 hemichannels and allows diffusion of small metabolites and cofactors through the β-cell plasma membrane. We have investigated in this islet “permeabilized” model whether glycolytic and citric acid cycle intermediates stimulate insulin secretion and how it correlates with ATP production (islet content plus extracellular nucleotide accumulation). Glycolytic intermediates (10 mM) stimulated insulin secretion and ATP production similarly. However, they showed differential sensitivities to respiratory chain or enzyme inhibitors. Pyruvate showed a lower secretory capacity and less ATP production than phosphoenolpyruvate, implicating an important role for glycolytic generation of ATP. ATP production by glucose-6-phosphate was not sensitive to a pyruvate kinase inhibitor that effectively suppressed the phosphoenolpyruvate-induced secretory response and islet ATP rise. Strong suppression of both insulin secretion and ATP production induced by glucose-6-phosphate was caused by 10 μM antimycin A, implicating an important role for the glycerophosphate shuttle in transferring reducing equivalents to the mitochondria. Five citric acid cycle intermediates were investigated for their secretory and ATP production capacity (succinate, fumarate, malate, isocitrate and α-ketoglutarate at 5 mM, together with ADP and/or NADP^+^ to feed the NADPH re-oxidation cycles). The magnitude of the secretory response was very similar among the different mitochondrial metabolites but α-ketoglutarate showed a more sustained second phase of secretion. Gabaculine (1 mM, a GABA-transaminase inhibitor) suppressed the second phase of secretion and the ATP-production stimulated by α-ketoglutarate, supporting a role for the GABA shuttle in the control of glucose-induced insulin secretion. None of the other citric acid intermediates essayed showed any suppression of both insulin secretion or ATP-production by the presence of gabaculine. We propose that endogenous GABA metabolism in the “GABA-shunt” facilitates ATP production in the citric acid cycle for an optimal insulin secretion.

## Introduction

According to the metabolic hypothesis, glucose has to be metabolized in the β-cells of pancreatic islets and increase the cytosolic ATP concentration and the ATP/ADP ratio in order to stimulate insulin secretion. The increased ATP/ADP ratio closes K_ATP_-dependent channels and depolarizes β-cells provoking in turn the opening of voltage-dependent Ca^2+^-channels and the elevation of the cytosolic cation concentration. The resulting stimulation of insulin secretion was called the “triggering phase” [1]. This phase may be reproduced at 5 mM glucose by β-cell membrane depolarization at a higher extracellular [KCl] than the physiological value, in the presence of diazoxide that keeps K_ATP_-channels open, and non-responsive to ATP. It results in a transient increase of insulin secretion lasting for about 10 minutes before returning to the basal secretory level [1]. The simultaneous presence of a stimulatory glucose concentration (6–20 mM) generates a second, sustained stimulation of insulin secretion of a magnitude dependent on the sugar concentration [1]. This second response was called the “amplifying phase” that is independent of K_ATP_-channels because they remain open due to the continuous presence of diazoxide.

Several candidates have been proposed as intracellular mediators of this second phase that would synergize the effect of the elevation of [Ca^2+^]_i_ on the exocytotic machinery: A sustained elevation of cytosolic ATP due to continuous glucose metabolism might be responsible for this synergism, independently of its effect of K_ATP_-channels [1]. NAD(P)H production [2–4] in several anabolic shuttles that exchange reducing power between mitochondria and cytosol has also been proposed as an alternative candidate for the amplification of the second phase acting on the exocytotic mechanism [5]. NAD(P)H may theoretically proceed from the three known mitochondrial shuttles where pyruvate is recycled: pyruvate-malate, pyruvate-citrate and pyruvate-isocitrate cycles [5]. Genetic suppression by adenovirus-mediated delivery of a specific siRNA against cytosolic or mitochondrial malic enzyme was without effect on glucose-induced insulin secretion in mouse islets [6]. This excludes the possibility that the two first proposed cycles (pyruvate-malate and pyruvate-citrate) are competent for the stimulation of insulin secretion by glucose [5]. However, genetic abrogation of the citrate/isocitrate carrier mediating the exchange of the two tricarboxylic acids across the inner mitochondrial membrane suppressed glucose-stimulation of insulin secretion in mouse islets [7]. Moreover, genetic suppression of cytosolic isocitric acid dehydrogenase by a specific siRNAs, mediated by infection of INS-1 cells with an adenovirus, induced a marked impairment of glucose-induced stimulation in INS-1cells and rat islets [8]. However, in a recent report, abrogation of cytosolic isocitric acid dehydrogenase by transfection of a specific siRNA targeting the same enzyme mRNA resulted in an improvement of insulin secretion [9]. In any case, suppression of the so called pyruvate/isocitrate [8] or pyruvate/α-ketoglutarate [9] cycle results in a decrease of the NADPH/ NADP^+^ ratio and the α-ketoglutarate concentration [8, 9]. In the pyruvate/isocitrate (α-ketoglutarate) cycle, isocitrate is finally exported into the cytosol and oxidized to α-ketoglutarate (αKG) and NADPH. No specific role has yet been attributed to the αKG generated that might be re-converted to pyruvate through the citric acid cycle pathway or, alternatively, participate in transamination reactions [5]. We have previously proposed that αKG generated during glucose metabolism in the citric acid cycle might accumulate due to the limiting rate of αKG-dehydrogenase and diversion into the “GABA-shunt” where it would be transaminated to glutamate and semialdehyde succinic acid [10]. The later would then be re-introduced into the citric acid cycle after being oxidized to succinic acid and NADH in the cytosol (semialdehyde succinic acid dehydrogenase (see ref. [11] for a scheme of GABA-shunt).

We have also previously demonstrated that extracellular ATP (millimolar range) is not only capable of triggering a second phase of insulin secretion in KCl-depolarized and “permeabilized” mouse and rat islets but also of potentiating the first phase independently of K_ATP_-channels [12, 13]. The main aim of this work was to investigate whether specific ATP producing pathways are linked to the stimulation of insulin secretion. Therefore, we have explored the secretory potential of glycolytic and citric acid cycle intermediates, accompanied by their essential metabolic cofactors, on the response of depolarized perifused rat islets “permeabilized” with 70 mM KCl. Intracellular ATP content and extracellular nucleotide accumulation were measured in parallel in incubated islets. It was also determined whether there is a regulation of the citric acid cycle metabolic flow by the availability of GABA. For that purpose, gabaculine (GABA-transaminase, GABA-T, inhibitor) was used to block the “GABA-shunt” coupled to the citric acid cycle fed by different cycle intermediates and observe the resultant consequences on ATP production (islet nucleotide content and its extracellular accumulation) and insulin secretion. These data supported important regulatory roles for glycolytic ATP production, the α-glycerophosphate shuttle and the GABA shunt in fuel-induced insulin secretion.

## Materials and Methods

### Materials

Collagenase P and FA-free bovine serum albumin were obtained from Roche Diagnostics S.L. (Barcelona, Spain). Bovine serum albumin and most of the substances (metabolites, cofactors, inhibitors) were obtained from Sigma-Aldrich Química S.A. (Madrid, Spain). Rat insulin standards were from Linco Research, Inc. (St. Charles, Missouri, U.S.A.). Na^125^I was obtained from PerkinElmer España, S.L. (Madrid, Spain). Inorganic compounds and organic solvents were obtained from VWR International Eurolab S.L. (Spain).

### Islet isolation and insulin secretion

Male Wistar-albino rats (250–275 g body weight) were obtained from the Complutense University colony. All animals were housed in cages at 22°C under a 12h light-dark cycle and fed ad libitum. Animal care, use, and experimental protocols were submitted and they were approved by the Ethics Committee of Complutense University, which was responsible for the correct application of Order 86/609/CEE (Spanish Order 1201/2005). Islets were isolated after collagenase digestion of the rat pancreas. Animals were sacrified by instantaneous decapitation after isoflurane anesthesia. The pancreas was rapidly dissected away, abdominal fat taken out, and the organ cut up in small pieces. Islets were isolated after collagenase digestion of the cut pancreas at 37°C for not more than 3–4 minutes. Insulin secretion was studied in perifused islets. Four groups, each of 40 collagenase-isolated islets, were perifused in parallel and at a flow rate of 0.5 ml/min with Krebs-Ringer, buffered with 0.5 mM NaHCO_3_ and 20 mM HEPES, supplemented with 0.5% FA-free BSA (KRBH), and heated at 37°C. The perifusion protocol was similar in all the experiments. After a pre-perifusion period of 45 minutes under basal conditions (in the absence of nutrients or at 5 mM glucose), the perifusion medium was switched to one containing the test substances and maintained for the next 30 (or 40) minutes. Finally, the medium was changed back to pre-perifusion conditions where it was maintained for the last 25 minutes. 70 mM KCl depolarization was always accompanied by addition of 250 μM diazoxide. The perifusate was collected at 1 minute intervals during the last 60–70 (80 in some experiments) minutes of perifusion and its insulin concentration was radioimmunologically measured. Pig insulin was radio-iodinated with Na^125^I [14] and rat insulin was used as a standard in the radioimmunoassay of insulin. Insulin antiserum was kindly provided by Dr. M. Villanueva-Peñacarrillo from the Department of Metabolism, Nutrition & Hormones, Fundación Jiménez Díaz, Madrid, Spain.

### Islet ATP-production: measurement of islet ATP-content and ATP-release (ATP accumulation in the incubation medium)

Three groups, each of 25 islets, were incubated at 37°C for 60 minutes in 25 μl of KRBH. An aliquot (20 μl) of the incubation medium was taken off and stored in clean tubes. The remaining medium was sucked off and islets washed thrice with KRBH containing 5 mM glucose and 50 μM mefloquine (connexin36 inhibitor in gap junction channels and hemichannels) [12, 13]: first, the islets were pipetted out of the tubes after addition of 100 μl of the washing solution; secondly, they were extensively washed in a Petri dish containing 5 ml washing solution; thirdly, the islets were washed again with 100 μl in a clean tube and finally suspended in 25 μl KRBH containing 5 mM glucose. The tubes containing the islets and those with the corresponding medium aliquots were frozen on acetone chilled with dry ice. 20 μl of 1.35 M perchloric acid (PCA) were then added, neutralized and the potassium perchlorate precipitated by centrifugation with 15 μl of 0.1 M Tris + 2.8 M of KHCO_3_. Clean supernatant aliquots (10 μl) of samples (from islets and medium for measurement of islet ATP-content and ATP-release, respectively) and ATP standard solutions (0–10 μM), treated in the same way, were mixed with 100 μl of D-luciferin solution (0.1 mM) in a 96 well plate. The emitted light was measured in a microplate reader (Synergy-2, Biotek) after addition of 10 μl of luciferase (0.1 mg/ml). No ATP-accumulation could be detected in the incubation medium with sufficient accuracy from intact, non-KCl-permeabilized islets incubated with or without metabolized substrates [13].

### Statistical analysis

Pairs of means ± S.E.M. were compared by Students’ t tests.

## Results

### Secretory capacity of glycolytic intermediates

**Fig 1:**
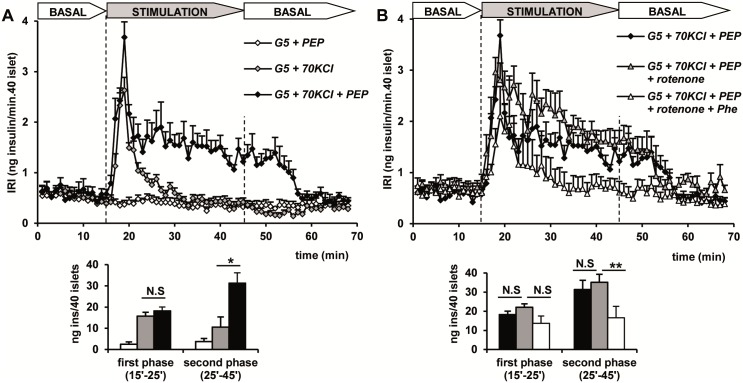
Ten mM phosphoenolpyruvate (PEP) stimulation of insulin secretion in depolarized (70 mM KCl = KCl) perifused rat islets and the effects of 10 μM rotenone (respiratory complex I inhibitor) and 15 mM phenylalanine (Phe) (pyruvate kinase inhibitor). (A) Groups of 40 islets each, pre-perifused with 5 mM glucose (G5) for 45 min, were stimulated for 30 min (between vertical broken lines) with 5 mM glucose (G5) + PEP under non-depolarizing (open symbols) or depolarizing conditions (KCl alone, gray symbols, or plus PEP, dark symbols). (B) Depolarized (G5 + KCl) islets were stimulated with PEP (+ 10 mM ADP) alone (dark diamonds) or together with rotenone (gray triangles) or rotenone + Phe (white triangles). Symbols and bars represent means ± S.E.M. of 6 experiments. (*p< 0.001 compared with the gray bar to the left; **p< 0.03 compared with the white bar to the right).

The secretory capacity of glycolytic intermediates participating in substrate-linked phosphorylation reactions was checked in rat islets permeabilized with 70 mM KCl and perifused at 5 mM glucose with the necessary additional cofactors for ATP synthesis. Perifusion with 10 mM phosphoenolpyruvate (PEP) together with 10 mM ADP did not stimulate insulin secretion in non-depolarized islets (Fig 1A). However, in 70 mM KCl-depolarized islets, PEP + ADP did not modify the transient peak due to KCl-depolarization alone but triggered a second phase of sustained insulin secretion that slowly returned to basal levels after withdrawing the metabolic stimulus (Fig 1A). Rotenone (10 μM, a respiratory complex I inhibitor) did not modify the response to PEP + ADP by itself and further addition of 15 mM phenylalanine (Phe, known pyruvate kinase inhibitor [15, 16]) suppressed the second phase of release by 53% (Fig 1B).

**Fig 2:**
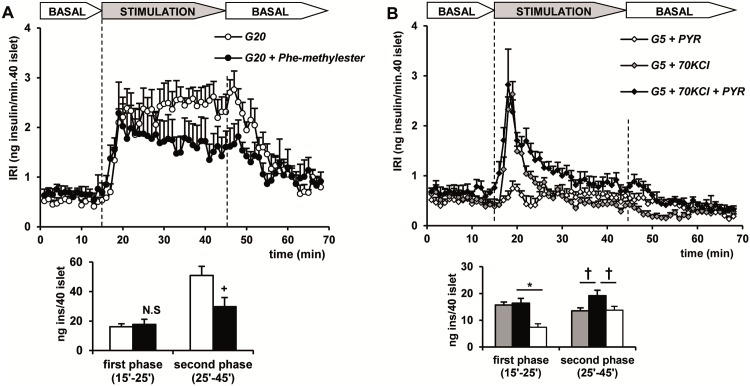
Effect of 15 mM phenylalanine methylester (Phen-methylester) on 20 mM glucose (G20) induced insulin secretion in control, non-depolarized islets (A) and secretory capacity of 10 mM pyruvate + 10 mM ADP (PYR) in depolarized (+KCl) and perifused islets. (A) Groups of 40 islets each, pre-perifused with 5 mM glucose (G5) for 45 min, were stimulated for 30 min (between vertical broken lines) with 20 mM glucose alone (open circles) or together with Phen-methylester. (B) Islets were stimulated with G5 + PYR under non-depolarizing (open diamonds) or depolarizing conditions (+KCl alone, gray diamonds; +KCl + PYR, dark diamonds). Symbols and bars represent means ± S.E.M. of 5 or 6 experiments (^+^p<0.04 compared with the white bar to the left; †p<0.05 compared with the white bar to the left).

As shown in Fig 2A, changing the glucose concentration of control (non-depolarized) islets from 5 to 20 mM stimulated a typical biphasic insulin secretion whose first phase was of similar magnitude to that triggered by 70 mM KCl depolarization. The second phase was, however, progressively increased and reached levels higher than the peak of the first phase and was also greater than the second phase induced by PEP + ADP under depolarizing conditions. 15 mM phenylalanine methylester (a permeable form of Phe, the pyruvate kinase inhibitor [15, 16]) suppressed the second phase of secretion stimulated by 20 mM glucose by 41% (Fig 2A). Fig 2B shows that 10 mM pyruvate (PYR), in the presence of 10 mM ADP, did not elicit any significant response in control islets with only a slight increase of the second phase in KCl-depolarized islets (p<0.05).

**Fig 3:**
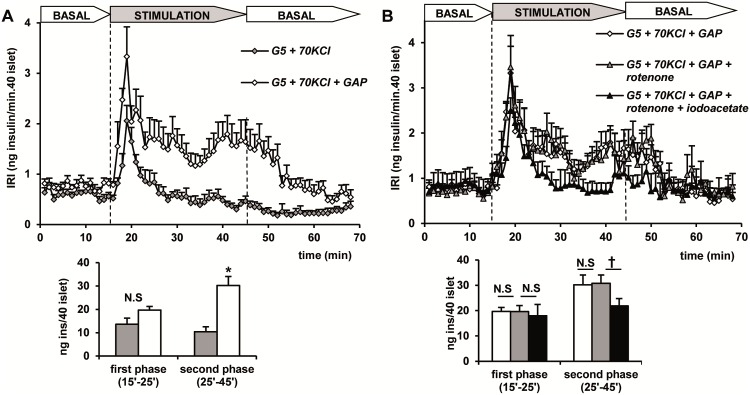
Secretory capacity of 10 mM glyceraldhyde-3-phosphate (GAP) together with 10 mM ADP and 10 mM NAD^+^ in depolarized (+KCl) islets and the effects of 10 μM rotenone (respiratory complex I inhibitor) and 0.5 mM iodoacetate (iodoacetate). (A) Groups of 40 islets each, pre-perifused with 5 mM glucose (G5) for 45 min, were stimulated for 30 min (between vertical broken lines) with G5 + KCl alone (gray diamonds) or together with GAP (with diamonds). (B) Islets were stimulated with G5 + KCl +GAP alone (white diamonds) or together with rotenone (gray triangles) or rotenone + iodoacetate (black triangles). Symbols and bars represent means ± S.E.M. of 5 or 6 experiments (*p<0.001 compared with the gray bar to the left; ^†^ p< 0.05 comparing the black with the gray bar.).

Glyceraldehyde-3- phosphate dehydrogenase (GAPDH) oxidizes its substrate with NAD^+^ as cofactor and generates 1, 3-diphospho glycerate that is sequentially coupled to ADP-phosphorylation in the next glycolytic reaction producing 3-phosphoglycerate plus ATP. Therefore, the secretory capacity of 10 mM glyceraldehyde-3- phosphate (GAP) was tested in perifused and depolarized islets in the presence of 10 mM ADP and 10 mM NAD^+^ (Fig 3A). Perifusion of GAP with only NAD^+^ did not modify KCl-induced secretion pattern at 5 mM glucose (results not shown). In the presence of only ADP, it significantly triggered a second phase of insulin secretion (p<0.07) (results not shown). However, the simultaneous perifusion of GAP with NAD^+^ and ADP trended to increase the first peak and induced a significant increment of the second (p<0.001) phase of release (Fig 3A). This metabolite mixture triggered a biphasic response of similar magnitude to that stimulated by PEP + ADP (see Fig 1A). Similarly to the latter stimulus, the secretory pattern induced by GAP (+NAD^+^+ ADP) was not affected by 10 μM rotenone but the second phase was suppressed by 0.5 mM iodoacetate (a GAPDH inhibitor) [17] by almost 30% in the presence of rotenone (Fig 3B).

**Fig 4:**
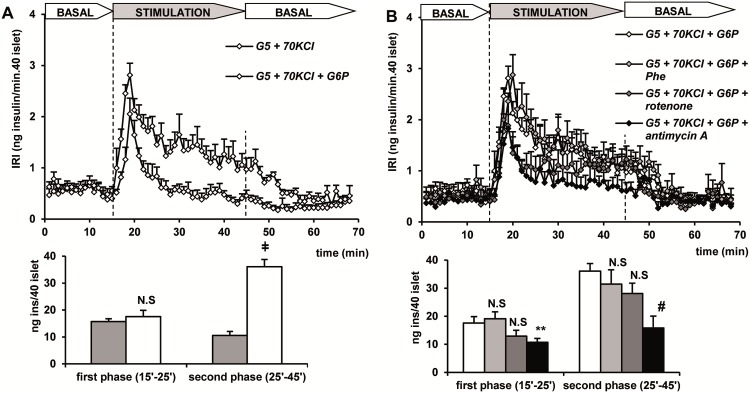
Secretory capacity of 10 mM glucose-6-phosphate (G6P) together with 10 mM ADP and 10 mM NAD^+^ in depolarized (+KCl) islets and the effects of 10 μM rotenone (respiratory complex I inhibitor), 10 μM antimycin A (respiratory complex III inhibitor) and 10 mM phenylalanine (Phe). (A) Groups of 40 islets each, pre-perifused with 5 mM glucose (G5) for 45 min, were stimulated for 30 min (between vertical broken lines) with G5 + KCl alone (white diamonds) or together with 10 mM G6P (gray diamonds). (B) Islets were stimulated with G5 + KCl + G6P alone (white diamonds) or together with Phen (light gray diamonds), rotenone (more intense gray diamonds) or antimycin A (black diamonds). Symbols and bars represent means ± S.E.M. of 6 experiments (p<0.0001 compared with the bar to left; **p<0.03 compared with the control white bar to the left; ^#^p<0.002 compared with the control white bar to the left).

Glucose-6-phosphate (G6P) at 10 mM, together with 10 mM NAD^+^ and 10 mM ADP, induced a second insulin secretory response in KCl-depolarized islets of similar magnitude to that previously shown for PEP or GAP (Fig 4A). The response triggered by G6P was not modified by 10 mM Phe and only slightly reduced by 10 μM rotenone. However, both the first (p<0.03) and second phases (p<0.002) of secretion were very significantly suppressed by 10 μM antimycin A (Fig 4B). The second phase due to G6P in the presence of the glycolytic cofactors was almost completely suppressed by 50 μM mefloquine (known connexin36 inhibitor in gap junction channels and hemichannels [12, 13]) (results not shown).

### Secretory capacity of citric acid cycle intermediates

It is believed that the stimulation of insulin secretion by glucose is not only the result of the ATP-dependent closure of K^+^-ATP channels but it also depends on specific effects of some glucose derived metabolites and/or cofactors produced in the citric acid cycle. It has been demonstrated that siRNA-mediated silencing of the cytosolic isoform of isocitric acid dehydrogenase is translated into inhibition of the insulin secretory response to glucose ultimately due to a decreased production of cytosolic α-ketoglutarate and/or NADPH in the pyruvate/isocitric cycle [8]. Therefore, it was the aim of this work to investigate the secretory effects of citric acid cycle metabolites, together with the cofactors implicated in their metabolism, in perifused rat islets permeabilized with 70 mM KCl.

**Fig 5:**
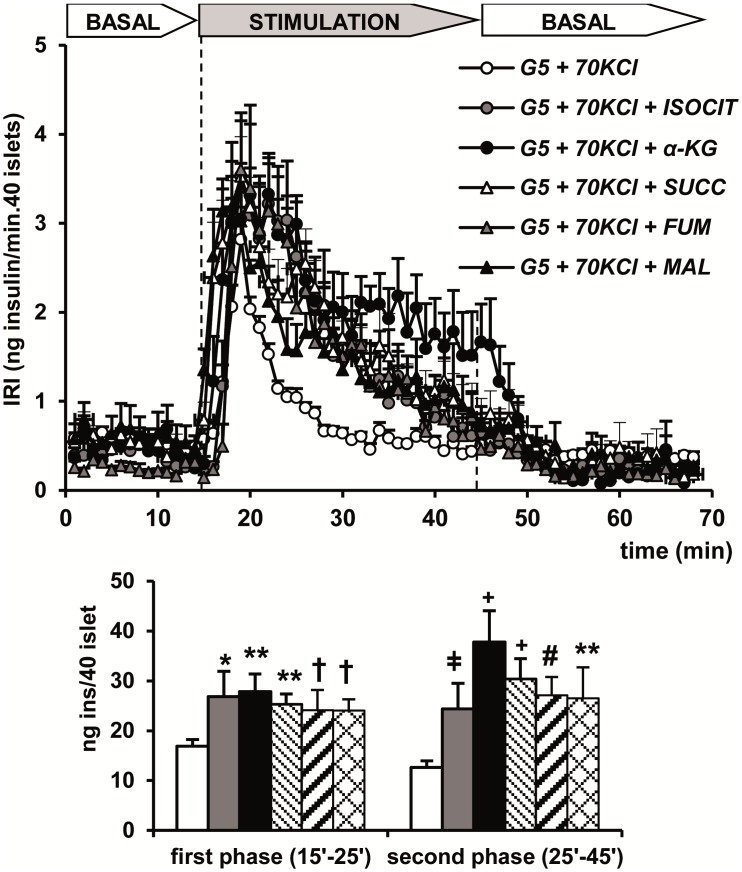
Secretory capacity of citric acid cycle intermediates at 5 mM (succinate, SUCC; fumarate, FUM; malate, MAL; isocitrate, ISOCIT; α-ketoglutarate, α-KG), together with the needed cofactors (5 mM ADP and/or 5 mM NADP) in perifused and depolarized (+ 70 mM KCl) islets. Groups of 40 islets each, pre-perifused with 5 mM glucose (G5) for 45 min, were stimulated for 30 min (between vertical broken lines) with G5 + KCl alone (white circle) or together with each of the citric acid cycle intermediates mentioned and the required cofactors (SUC+ADP+NADP^+^; FUM+ADP; MAL+ADP+NADP^+^; α-KG+ADP+NADP^+^; ISOCIT+ADP+NADP^+^). Symbols (see the insert in the Figure) and bars represent means ± S.E.M. of 5 experiments (*p<0.01, **p<0.03, ^†^p<0.03, p<0.004 and ^#^p<0.002 compared with the first white bar to the left).

Fig 5 compares the secretion profiles induced by 5mM α-ketoglutarate (+5 mM ADP), 5 mM succinate (+5 mM ADP and 5 mM NADP^+^), 5 mM fumarate (+ 5 mM ADP), 5 mM malate (+5 mM ADP and 5 mM NADP^+^), and 5 mM isocitrate (+5 mM ADP and 5 mM NADP^+^) in perifused rat islets permeabilized with 70 mM KCl. All five metabolic intermediates increased the first peak of secretion induced by KCl depolarization alone and triggered a second phase of insulin release above the levels obtained with depolarization alone that progressively declined with time (Fig 5). However, no significant differences were found among the secretory capacities of the five intermediates as measured by the amount of insulin released in either the first (10 min) or the second (subsequent 20 min) phase of secretion. Strikingly, α-ketoglutarate showed a more sustained second phase of secretion (20 min) that did not reach quantitative significance compared with the other citric acid cycle intermediates, probably due to the short perifusion time (20 min. for the second phase of secretion).

**Fig 6:**
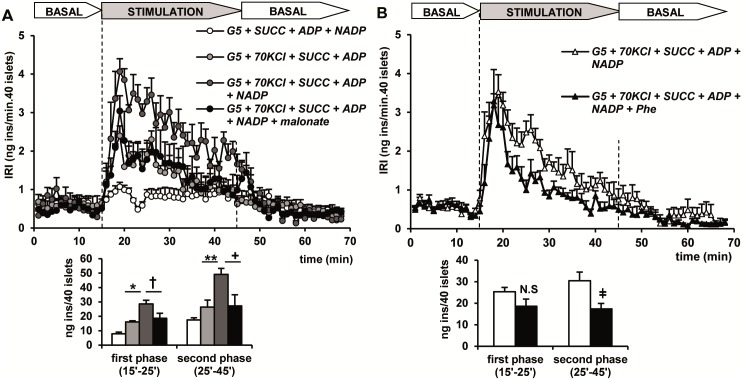
Characterization of the capacity to stimulate insulin secretion of 5 mM succinate (SUCC) and the cofactors required for the pyruvate/malate cycle (5 mM ADP and 5 mM NADP^+^) in control and depolarized (+KCl) islets. Effects of 5 mM malonate (succinate dehydrogenase inhibitor) and 15 mM Phe (PK inhibitor). (A) Groups of 40 islets each, pre-perifused with 5 mM glucose (G5) for 45 min, were stimulated for 30 min (between vertical broken lines) with G5 + KCl together with SUCC+ADP, SUCC+ADP+NADP^+^, SUCC+ADP+ NADP^+^+5 mM malonate or with G5 (at physiological KCl concentration) together with SUCC+ADP+ NADP^+^ (see the corresponding symbols in the insert of the Figure). (B) Depolarized islets (+KCl) were stimulated with SUCC+ADP+NADP^+^ alone (white triangles) or toghether with 15 mM Phen (dark triangles). Symbols (see the insert in the Figure) and bars represent means ± S.E.M. of 5 experiments (*p<0.02, **p<0.007, ^†^p<0.05, ^+^ p<0.04 and p<0.02 compared with the first white bar to the left).

The secretory capacity of succinate in depolarized islets was investigated in more detail to check its dependence on mitochondrial metabolism. First of all, 5 mM succinate (together with 5 mM ADP to maintain a high rate of oxidative phosphorylation, plus 5 mM NADP^+^ to allow flow through the pyruvate / malate cycle and generation of cytosolic NADPH and pyruvate) was tested in control, non-depolarized islets, perifused at a physiological KCl concentration (4, 7 mM). No significant elevation above the basal rate of secretion was observed (Fig 6A). In islets depolarized with 70 mM KCl, succinate plus ADP alone stimulated a first and second phase of insulin secretion (Fig 6A). Further addition of NADP^+^, increased both phases of the secretory response triggered by succinate in the sole presence of ADP (Fig 6A). It was determined if a cytosolic increase of NADPH (in the absence of NADP^+^) might potentiate ATP stimulated insulin secretion in depolarized islets: 1 mM NADPH had no synergistic effect on the stimulation of the first (19.1 ± 2.4, n = 6, vs. 22.0 ± 3.0 ng /40 islets x 10 min, n = 5; NS) or second phase of release by 10 mM extracellular ATP (31.6 ± 6.6, n = 5, vs. 27.5 ± 2.4 ng/40 islets x 20 min, n = 6; NS) (results not shown). Five mM malonate, a known inhibitor of succinic acid dehydrogenase [18], significantly decreased the first (p< 0.049) and second phase (p< 0.04) of secretion induced by the metabolic mixture of succinate with ADP and NADP^+^ in depolarized islets (Fig 6A). Fifteen mM Phe (a known pyruvate kinase inhibitor) [15, 16] slightly decreased the first peak and more consistently diminished the second phase of the insulin response of depolarized islets to succinate in presence of ADP and NADP^+^ (Fig 6B).

### Interactions between the citric acid cycle and the “GABA-shunt”

**Fig 7:**
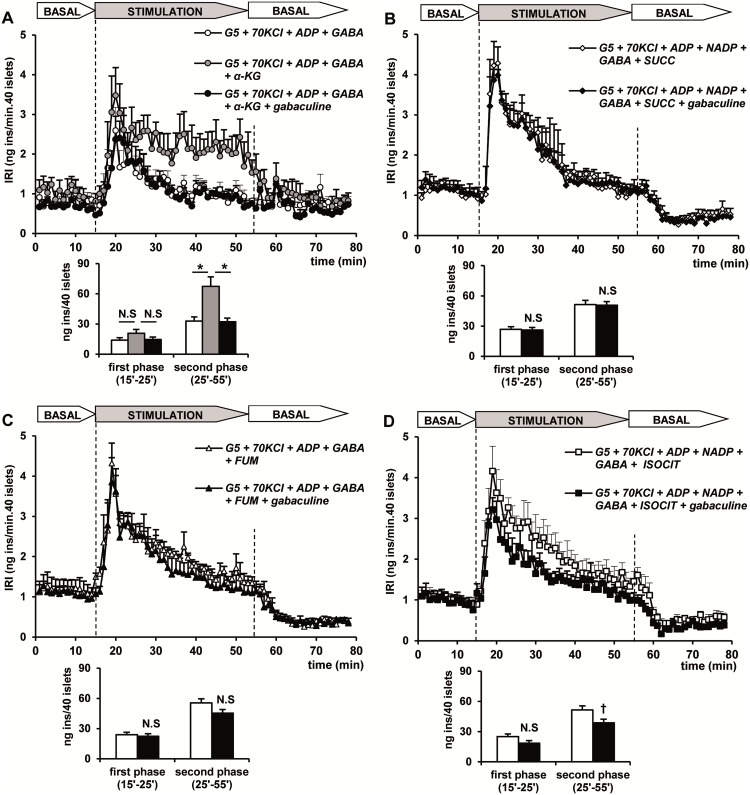
Effect of 1 mM gabaculine (GABAT inhibitor) on the stimulation of insulin secretion by citric acid cycle intermediates at 5 mM (succinate, SUCC; fumarate, FUM; isocitrate, ISOCIT; α-ketoglutarate, α-KG), together with 2 mM GABA (GABA) and 5 mM of required cofactors (ADP and/or NADP^+^) in depolarized (+70 mM KCl, KCl) islets. (A) Groups of 40 islets each, pre-perifused with 5 mM glucose (G5) for 45 min, were stimulated for 40 min (between vertical broken lines) with G5+KCl together with ADP+GABA (open circles, n = 5), α-KG+ADP+GABA (gray circles, n = 8) or α-KG+ADP+GABA +gabaculine (dark circles, n = 10). (B) Islets were depolarized (+70 mM KCl, KCl) and stimulated with SUCC+ADP+NADP^+^+GABA in the absence (white diamonds, n = 5) or presence of gabaculine (dark diamonds, n = 5). (C) Islets were depolarized (+70 mM KCl, KCl) and stimulated with FUM+ADP+GABA in the absence (white triangles, n = 4) or presence of gabaculine (dark triangles, n = 4). (D) Islets were depolarized (+70 mM KCl, KCl) and stimulated with ISOCIT+ADP+NADP^+^+GABA in the absence (white squares, n = 4) or presence of gabaculine (dark squares, n = 4). Symbols (see the insert in the Figure) and bars represent means ± S.E.M. of n experiments (given above within parenthesis) (*p<0.02 compared with the white bar to the left or the dark bar to the right; ^†^p<0.05 compared with the white bar to the left).

The dependence of the stimulation of insulin secretion by citric acid cycle intermediates on “GABA-shunt” metabolism was next evaluated in 70 mM KCl-depolarized islets. Two mM extracellular GABA was added to compensate for islet γ-amino acid loss induced by depolarization [11, 12] and GABA-transaminase (GABA-T) was inhibited with 1 mM gabaculine [19]. The average rate of insulin secretion during the second phase stimulated by the citric acid cycle intermediates (α-ketoglutarate, succinate, fumarate and isocitrate) assayed in the presence of 2 mM GABA was statistically higher than in the absence of the γ-amino acid (1.77 ± 0.04 ng/min, n = 4, vs. 1.43 ± 0.17 ng/min, n = 4; p< 0.007). Note that in all the following islet perifusion experiments, the stimulatory phase of secretion was increased from 30 to 40 min in order to better discriminate whether the second phase of insulin secretion was sustained or not. Fig 7A shows that 5 mM α-ketoglutarate, together with 5 and 2 mM ADP and GABA, respectively, stimulated a biphasic secretion of insulin. It did not modify the first phase due to KCl-depolarization but induced a second and sustained phase. Gabaculine (1 Mm) slightly reduced the first phase but suppressed the second phase by 47.4% (Fig 7A). Like α-ketoglutarate, both succinate and fumarate, with their corresponding cofactors (ADP and NADP^+^ and ADP, respectively) and 2 mM GABA, also stimulated a second phase of insulin secretion of similar magnitude that progressively declined to a lower steady value. At variance with α-ketoglutarate, the second phase stimulated by succinate or fumarate was not sensitive to 1 mM gabaculine (Fig 7B and 7C). Isocitrate, in the presence of ADP and NADP^+^, similar to the other assayed intermediates, triggered a second phase of secretion after the first peak induced by KCl-depolarization (Fig 7D). However, at variance with succinate and fumarate, the isocitrate-induced second phase was significantly suppressed by 1 mM gabaculine although not so strongly as in the case of α-ketoglutarate (Fig 7D).

### ATP production by glycolytic intermediates in KCl-depolarized islets

It has been clearly demonstrated previously that KCl-depolarization markedly decreases islet ATP content that can be reversed adding extracellular ATP [11, 12]. It has been assessed in this work whether glycolytic and/or mitochondrial metabolites might generate ATP and increase its concentration in KCl-permeabilized islets. It was quickly realized that the ATP produced by glycolytic reactions coupled to ADP phosphorylation not only increased islet ATP-content but diffused out of the KCl-permeabilized islet β-cells and accumulated extracellularly. Therefore, ATP was measured both in the tissue (washed islets after 1 h incubation) and in an aliquot of the incubation medium (described in more detail in the Section of Materials and Methods). The amount of ATP released and accumulated in the incubation medium was approximately 1,000-fold higher than in the tissue (25 rat islets in 25 μL). However, due to the high ectonucleotidase activity in the membrane of islet cells [12] the measurement of accumulated extracellular ATP was not completely accurate and likely a close underestimation of the true value.

In control, non-depolarized islets, 10 mM PYR and PEP, together with 10 mM ADP and 5 mM glucose, did not increase the islet ATP content above the value regularly found at 5 mM glucose alone (2.70 ± 0.3 pmol/ islet, n = 5, and 2.28 ± 0.20 pmol/islet, n = 6, respectively, vs. 2.8 ± 0.14, n = 18; NS). Low nanomolar amounts of ATP were simultaneously recorded in the corresponding incubation media (0.007 ± 0.02, n = 5, and 0.006 ± 0.02 nmol/islet, n = 6) of islets incubated with PYR or PEP, respectively.

All the glycolytic intermediates tested in KCl-depolarized islets (G6P, GAP, PEP and PYR together with the corresponding cofactors as described above in the paragraph entitled: **Secretory capacity of glycolytic intermediates**) increased islet ATP content to the maximum values recorded in the presence of 20 mM glucose (4–5 pmol/islet) and they were strongly suppressed in the presence of 10 μM antimycin A (inhibitor of respiratory complex III) (Table 1). Simultaneously, a similar increase of the extracellular ATP accumulation (ATP-released, between 5–6 nmol / islet) was recorded with all the glycolytic metabolites except PYR (Table 1). The latter (at 10 mM) induced significantly less (-33%) accumulation of extracellular ATP than 10 mM PEP (lower half of Table 1: 3.62 ± 0.42, n = 5 vs. 5.4 ± 0.47 nmol /islet, n = 5; p<0.022). Antimycin A (10 μM) significantly reduced the increase in the extracellular ATP generated by the metabolism of G6P and GAP but not of PYR and PEP (Table 1). In contrast, 10 μM rotenone (inhibitor of respiratory complex I) strongly suppressed the elevation of islet ATP content induced by G6P, GAP, PEP and PYR but it did not affect the extracellular accumulation of ATP (Table 1). Fifteen mM Phe significantly suppressed both the content of ATP (2.24 ± 0.52, n = 4 vs 5.18 ± 0.73 pmol/islet, n = 5; p<0.02) and its extracellular accumulation (3.23 ± 0.36, n = 5 vs 5.40 ± 0.47 nmol/islet, n = 5; p<0.01) in KCl-depolarized islets incubated with 10 mM PEP and ADP.

**Table 1:** Measurement of ATP production by islets depolarized with 70 mM KCl: islet ATP content and its accumulation in the extracellular medium in response to addition of the indicated glycolytic intermediate and its corresponding cofactors. Effects of the complex respiratory inhibitors 10 μM rotenone and 10 μM antimycin A. Three groups, each of 25 islets, were incubated at 37°C for 60 minutes in 25 μl of KRBH. At the end of incubation, medium samples were separated and stored and islets consistently washed to dilute the contaminating extracellular nucleotide. After addition of perchloric acid to medium and islet samples, they were neutralized and potassium perchlorate precipitated. ATP was measured in samples of the clean supernatant with the luciferin/luciferase system. The emitted light was measured in a microplate reader (Synergy-2, Biotek) after addition of luciferase. More detailed information can be found in the Materials and Methods section.

	ATP content (pmol ATP/islet)			ATP medium (nmol ATP/islet)		
		**+ antimycin A**	***p***		**+ antimycin A**	***p***
**10 mM G6P**	4.89 ± 0.68 (4)	1.73 ± 0.23 (5)	<0.002	5.13 ± 0.56 (5)	3.07 ± 0.11 (5)	<0.01
**5 mM GAP**	4.29 ± 0.45 (5)	1.20 ± 0.37 (5)	<0.0005	6.37 ± 0.93 (5)	3.97 ± 0.21 (5)	<0.04
**5 mM PEP**	4.14 ± 0.25 (5)	1.23 ± 0.42 (5)	<0.0005	5.07 ± 0.57 (5)	4.73 ± 0.72 (5)	N.S.
**10 mM PYR**	4.60 ± 0.55 (5)	1.65 ± 0.17 (6)	<0.001	3.62 ± 0.42 (5)	3.06 ± 0.12 (5)	N.S.
		**+ rotenone**	***p***		**+ rotenone**	***p***
**10 mM G6P**	4.89 ± 0.68 (4)	1.83 ± 0.43 (5)	<0.005	5.13 ± 0.56 (5)	4.46 ± 0.66 (5)	N.S.
**10 mM GAP**	4.29 ± 0.60 (5)	1.05 ± 0.11 (5)	<0.001	6.09 ± 1.04 (5)	5.87 ± 0.08 (5)	N.S.
**10 mM PEP**	5.18 ± 0.73 (5)	1.76 ± 0.21 (5)	<0.002	5.40 ± 0.47 (5)	5.47 ± 0.71 (5)	N.S.
**10 mM PYR**	4.60 ± 0.55 (5)	1.36 ± 0.23 (6)	<0.001	3.62 ± 0.42 (5)	-	

### Islet ATP-content and ATP-release in the incubation medium induced by citric acid cycle intermediates in KCl-depolarized islets

All tested metabolites (isocitrate, α-ketoglutarate, succinate, fumarate and malate), assayed at 5 mM with their corresponding cofactors, raised intracellular ATP to similar levels as 20 mM glucose in KCl-depolarized islets (around 4 pmol /islet; Table 2). There was a similar accumulation of extracellular ATP in depolarized islets incubated with α-ketoglutarate and succinate. However, fumarate and isocitrate generated a significantly lower accumulation of extracellular ATP than α-ketoglutarate (Table 2: *p<0.0006 and **p<0.005, respectively). Fifteen mM Phe did not modify either the islet ATP content (4.57 ± 0.35, n = 9 vs 5.09 ± 0.40 pmol/islet, n = 5; N.S.) or its extracellular accumulation (4.10 ± 0.30, n = 10 vs 3.59 ± 0.30 nmol/islet, n = 5; N.S.) in islets incubated with succinate. In contrast, 10 μM Antimycin A strongly diminished ATP content in depolarized islets incubated with either succinate or malate but not the extracellular nucleotide accumulation (Table 2). Malonate (10 mM), a competitive inhibitor of succinic acid dehydrogenase, significantly decreased both the islet content of ATP (2.47 ± 0.45, n = 5 vs 4.23 ± 0.55 pmol/islet, n = 4; p<0.02) as well as its extracellular accumulation (3.14 ± 0.46, n = 5 vs 4.96 ± 0.38 nmol/islet, n = 4; p<0.02) in depolarized islets incubated with 10 mM succinate (and the cofactors ADP and NADP^+^ at 10 mM)

**Table 2:** Measurement of ATP production by islets depolarized with 70 mM KCl: islet ATP content and its accumulation in the extracellular medium in response to addition of the indicated citric acid cycle intermediate and its corresponding cofactors for the pyruvate/malate or isocitrate/malate cycles. Effect of 10μM antimycin A. The experimental conditions were the same as described above in the legend of Table 1. (More information is found in Materials and Methods section).

	ATP content (pmol ATP/islet)			ATP medium (nmol ATP/islet)		
		+ antimycin A	*p*		+ antimycin A	*p*
**5 mM ISOCIT**	3.96 ± 0.55 (4)	-	-	2.76 ± 0.25 (4)**	-	-
**5mM α-KG**	4.11 ± 0.47 (4)	-	-	4.65 ± 0.35 (4)	-	-
**5mM SUCC**	4.57 ± 0.35 (9)	1.36 ± 0.57 (5)	<0.0002	4.10 ± 0.30 (10)	3.89 ± 0.41 (5)	N.S.
**5mM FUM**	3.85 ± 0.58 (4)	-		2.30 ± 0.07 (4)*	-	
**5mM MAL**	4.69 ± 0.49 (5)	1.72 ± 0.46 (5)	<0.002	3.81 ± 0.23 (5)	3.48 ± 0.50 (5)	N.S.

(*p<0.0006 and **p<0.005 compared with the amount of medium ATP accumulated by α-KG)

For reasons mentioned above (**Interactions between the citric acid cycle and the “GABA-shunt”**), it was determined whether islet ATP production by citric acid cycle intermediates (isocitrate, α-ketoglutarate, succinate and fumarate) in KCl-depolarized islets was sensitive to 1 mM gabaculine, a GABA transaminase inhibitor. To compensate for the loss of islet GABA due to KCl depolarization [11, 13], 2 mM extracellular GABA was added in all the experiments. The presence of the γ-amino acid increased islet ATP content induced by the four metabolic intermediates (succinate, fumarate, isocitrate and α-ketoglutarate) but it reached only statistical significance with fumarate (3.85 ± 0.48, n = 4 vs 5.31 ± 0.24, n = 5 pmol/islet; p<0.05). However, 2 mM GABA increased the average islet ATP released in the medium by the four tested metabolic intermediates (succinate, fumarate, isocitrate and α-ketoglutarate) (4.9 ± 0.18, n = 4, vs. 4.0 ± 0.12 nmol/islet, n = 4; p<0.008). Gabaculine (1 mM) significantly suppressed the rise in islet ATP-content induced by 5 mM α-KG by -25% and the extracellular ATP accumulation by -37% (Table 3). The drug affected, however, neither islet ATP content nor ATP-medium accumulation by any of the other citric acid cycle intermediates assayed.

**Table 3:** Measurement of ATP production by islets depolarized with 70 mM KCl: islet ATP content and its accumulation in the extracellular medium in response to addition of the indicated citric acid cycle intermediate, its corresponding cofactors for the pyruvate/malate or isocitrate/malate cycles, and 2 mM GABA. Effect of the GABAT inhibitor 1 mM gabaculine. The experimental conditions were the same as described above in the legend of Table 1. (More information is found in Materials and Methods section).

	ATP content (pmol ATP/islet)			ATP medium (nmol ATP/islet)		
	+ 2 mM GABA			+ 2 mM GABA		
		+ gabaculine	*p*		+ gabaculine	*p*
**5 mM ISOCIT**	4.84 ± 0.44 (5)	3.86 ± 0.25 (4)	N.S.	3.83 ± 0.45 (5)	3.02 ± 0.37 (5)	N.S.
**5mM α-KG**	4.78 ± 0.50 (8)	3.57 ± 0.28 (7)	<0.05	4.32 ± 0.29 (8)	2.72 ± 0.23 (9)	<0.0005
**5mM SUCC**	4.45 ± 0.42 (4)	4.18 ± 0.63 (4)	N.S.	3.83 ± 0.36 (4)	3.49 ± 0.16 (4)	N.S.
**5mM FUM**	5.31 ± 0.24 (5)	4.87 ± 0.15 (5)	N.S.	4.11 ± 0.23 (5)	3.80 ± 0.41 (5)	N.S.

## Discussion

The current work demonstrated that feeding glycolytic intermediates to metabolic reactions catalyzing substrate-linked ADP phosphorylation in KCl-permeabilized islets induced a second phase of insulin secretion subsequent to the first phase induced by KCl itself. Extracellular PEP + ADP stimulated a second phase of secretion that returned to basal values following withdrawal of the metabolic stimulus that was accompanied by an increased islet content of ATP and its release and accumulation in the incubation medium. As we have demonstrated previously [12, 13], ATP release or uptake in KCl “permeabilized” islets is due to diffusion of the nucleotide through open Cx36-hemichannels. The continuous availability of ADP together with a specific substrate for a “substrate-linked phosphorylation” or a citric acid cycle metabolite generated a continuous synthesis of ATP that raised its intra- and extra-cellular concentrations considerably. The source of ATP accumulated in the medium is probably both cytosolic and mitochondrial, the proportion depending on the substrate. The accumulation of extracellular ATP is perhaps a more quantifiable measurement of the total amount of ATP produced than the elevation of the islet content. It seems reasonable to assume that the higher the extracellular ATP accumulation, the higher the cytosolic ATP concentration. We attribute the stimulation of insulin secretion by PEP+ADP mainly to an increased cytosolic ATP-content or concentration generated by pyruvate kinase. In fact, 15 mM Phe (a known PK inhibitor) strongly suppressed PEP-stimulated secretion. It was not affected by 10 μM rotenone (respiratory complex I inhibitor) even though islet ATP content was markedly reduced but medium ATP accumulation was not affected. Antimycin A (respiratory complex III inhibitor) also decreased islet ATP content but not medium ATP accumulation stimulated by PEP + ADP. Therefore, it might be concluded that PEP stimulation of a second phase of insulin secretion in depolarized islets was due predominantly to an increased cytosolic ATP production in the PK reaction. The resulting pyruvate was probably metabolized in the mitochondria but seemed to contribute little to the stimulation of insulin secretion: rotenone was without effect even though it markedly suppressed PEP induced elevation of islet ATP content probably mediated by pyruvate metabolism. This is supported by the observation that exogenous PYR + ADP (both at 10 mM) only slightly stimulated insulin secretion despite increasing islet ATP content to similar levels as PEP that were also, similarly, strongly decreased by 10 μM rotenone and antimycin A. However, the amount of ATP-released in the medium by PYR was smaller than that induced by PEP. It has been concluded after mass-isotopomeric flux analysis of glycolytic flux in insulinoma INS-1 cells that anaplerotic carboxylation of pyruvate represents only a minority of the overall citric acid cycle and that the carbon flow from pyruvate to oxaloacetate/malate and back to PEP/pyruvate does not mix with glycolytic pyruvate [20]. It might explain the low secretory capacity of exogenously added pyruvate as due to its poor metabolism in the citric acid cycle because of a limitation of the cycle anaplerotic replenishment under the prevalent experimental conditions. By contrast, it was verified that PK inhibition with 15 mM phenylalanine methylester (a membrane permeable form of Phe) [15, 16] significantly suppressed (-41%) the second phase of insulin secretion stimulation by 20 mM glucose in control, non-depolarized islets. This again supports that ATP synthesized in the PK reaction is mainly responsible for the stimulation of insulin secretion by PEP in “KCl-permeabilized” islets. The magnitude of the second phase of the secretory response to glucose was always higher than that stimulated in depolarized islets by any of the other glycolytic or citric acid cycle metabolites. KCl-induced β-cell membrane permeability at low glucose probably modified the intracellular composition (partial loss of metabolites and cofactors) so that the system had a reduced maximum capacity to secrete insulin but it was still capable of responding reversibly to metabolic stimuli (extracellular metabolites plus cofactors).

Glyceraldehyde-3-phosphate (GAP) and glucose-6-phosphate (G6P), both at 10 mM together with the cofactors 10 mM ADP and 10 mM NAD^+^, stimulated a second phase of insulin release in “KCl-permeabilized” islets of similar magnitude to that of PEP + ADP. Similarly to PEP, the secretory response to GAP (+ ADP + NAD^+^) was not significantly affected by 10 μM rotenone that, however, suppressed islet ATP content but not its accumulation in the medium. At variance with PEP, 10 μM antimycin A (respiratory complex III inhibitor) suppressed not only islet ATP content elevated by GAP but also its extracellular accumulation. Given the relatively poor contribution of pyruvate metabolism to insulin secretion in “permeabilized” islets, the main sources of ATP for secretion might be the mitochondrial re-oxidation of cytosolic NADH (glycerophosphate shuttle) and PEP-linked ADP-phosphorylation. Inhibition of GAP-induced secretion by 0.5 mM iodoacetate (GAPDH inhibitor) supported attribution of its effect on metabolism of the triose phosphate. Similarly to PEP and GAP, the second phase of insulin release induced by G6P was not changed by 10 μM rotenone that only suppressed islet ATP content but not its extracellular accumulation. However, in common with GAP, 10 μM antimycin A suppressed both islet ATP content and its medium accumulation. Moreover, in this case it was demonstrated that 10 μM antimycin A significantly decreased both the first and second phase of the stimulation of insulin release by G6P. As 10 mM Phe did not modify the G6P secretory response, the most relevant source of ATP for secretion when the whole glycolytic flow is increased in this “permeabilized” islet model seems to be mitochondrial re-oxidation of cytosolic NADH generated at the GAPDH step by the α-glycerophosphate shuttle. This is supported by a previous study demonstrating that β-cells express a higher level of mitochondrial glycerolphosphate dehydrogenase than cytosolic lactate dehydrogenase [21]. As a main conclusion, islet glycolysis needs the participation of mitochondrial oxidative phosphorylation to produce the amount of ATP needed for a sustained stimulation of insulin secretion.

All five citric acid cycle intermediates tested (succinate, fumarate, malate, isocitrate and α-ketoglutarate, each at 5 mM), accompanied by the required cofactors (NADP^+^ and/or ADP, each at 5 mM), stimulated a second phase of insulin secretion in depolarized islets and potentiated the first phase induced by KCl alone. There was no significant difference among the magnitude of the stimulated second phases but the response to α-ketoglutarate was more sustained compared to other intermediates. Each of them increased islet ATP content to stimulatory values similar to those reached with 20 mM glucose that were strongly suppressed by 10 μM antimycin A when tested on succinate and malate. However, the extracellular ATP accumulation differed among the citric acid cycle intermediates but it was nevertheless increased by their presence (Table 2). This seems to be at variance with the lack of response to glucose of oligomycin-dependent oxygen consumption in INS-1 cells, attributed to an increased mitochondrial proton leak [20]. If this were to apply also to primary β-cells, an increased glycolytic production of ATP might, alternatively, be more specifically linked to the stimulation of insulin secretion by glucose. The magnitude of the secretory response to succinate and ADP was further potentiated by NADP^+^ suggesting that the pyruvate/malate cycle was operative and produced pyruvate that was partially metabolized in the mitochondria. Malonate (inhibitor of succinic acid dehydrogenase) suppressed both phases of the secretory response supporting the correspondence between citric acid cycle metabolism and insulin secretion in “permeabilized” islets, as it has been previously confirmed in control, non-depolarized, islets [18]. The inhibition of the second phase of the insulin response to succinate by Phe reinforces the observation that PEP is produced in the citric acid cycle and exported to cytoplasm where it can serve as substrate of PK and directly generate cytosolic ATP [15]. One mM NADPH, alone, did not modify either 70 mM KCl-induced insulin secretion or the stimulation of a second phase stimulated by 10 mM ATP. These results are at variance with the potentiation by 0.1 mM NADPH of membrane capacitance elicited primarily with depolarization pulses in β-cells clamped in the whole cell configuration [22]. Perhaps this difference can be attributed to differences in the cytosolic ATP concentration between the β-cell clamped model (3 mM) and our “permeabilized” islet preparation exposed to 10 mM extracellular ATP that might elevate nucleotide levels to a maximum value for stimulation [13].

Strong support for a role of the islet “GABA-shunt” is based on evidence that provision of α-ketoglutarate during glucose metabolism in the cytric acid cycle enhances insulin secretion [19]. The “GABA-shunt” is sensitive to inhibition by the GABA transaminase specific inhibitor, gabaculine [19] that was used to document the dependence of GABA mitochondrial metabolism on α-ketoglutarate production [19], or, in the present paper, the dependence of insulin secretion induced by citric acid cycle metabolites on “GABA-shunt” metabolism. We have shown that α-ketoglutarate stimulated a clearly sustained second phase of insulin secretion in “permeabilized” islets and the presence of 2 mM GABA, in contrast to a declining later phase induced by succinate, fumarate or isocitrate. One mM gabaculine markedly suppressed by 47.4% and 25% the second phase of secretion stimulated by α-ketoglutarate and isocitrate, respectively; it had no significant effect on the insulin response stimulated by either succinate or fumarate that enter the citric acid cycle further away from α-ketoglutarate. The GABA transaminase inhibitor also diminished ATP production by α-ketoglutarate, both islet ATP-content and its extracellular accumulation by 25 and 37%, respectively. It seems plausible that the gabaculine inhibition of α-ketoglutarate stimulation of insulin secretion in “permeabilized” islets was secondary to the suppression of cellular ATP production caused by “GABA-shunt” inhibition. Gabaculine also inhibits 20 mM glucose induced insulin secretion and the elevation of islet ATP and the ATP/ADP ratio in control, non-depolarized, islets [10]. We interpret this to a low activity of α-ketoglutarate dehydrogenase that limits metabolic flow in the citric acid cycle. This limitation might be released by the availability of GABA that would divert α-ketoglutarate into the “GABA-shunt” and convert it to succinate. Islet expression profiles of some citric acid cycle and “GABA-shunt” enzymes showed that αKGDH was the least expressed mRNA: GABA transaminase activity is almost 3-fold higher than αKGDH in islet homogenates ([10], Suppl. Fig.S4). Islet or purified β-cell GABA-content should change inversely with the flow rate in the citric acid cycle and this fits into the experimental evidence that the γ-amino acid content decreases with elevation of the glucose concentration and the corresponding stimulation of insulin release [10, 23]. Its function might be to facilitate an increase of the metabolic rate in the citric acid cycle in response to elevated glucose. Suppression of islet “GABA-shunt” metabolism might specifically reduce the contribution of mitochondrial ATP-production to the stimulation of secretion. To our knowledge, it is not known whether blocking “GABA-shunt” metabolism results in a suppression of β-cell glycolysis. However, there is old but well compelling evidence that inhibition of mitochondrial respiration by anoxia or respiratory inhibitors results in a suppression of the rate of ^3^H_2_O-production from stimulatory concentrations of D-[5-^3^H]glucose [21, 24, 25].

In summary, our data document three major sites where increased ATP production can enhance insulin secretion. As noted above, the “GABA-shunt” can participate by bypassing a citric acid cycle limitation at α-ketoglutarate dehydrogenase. In addition, flux though the α-glycerophosphate shuttle can contribute by promoting the re-oxidation of glycolytic NAD^+^, which limits flux through glyceraldehyde 3-phosphate dehydrogenase. Finally, pyruvate kinase, the last step in glycolysis was shown to be a major site of ATP production linked to insulin secretion. All three sites are likely to participate in response to physiological fuel stimulation and could play a regulatory role under conditions of high demand.
